# Drivers of avian habitat use and detection of backyard birds in the Pacific Northwest during COVID-19 pandemic lockdowns

**DOI:** 10.1038/s41598-022-16406-w

**Published:** 2022-08-11

**Authors:** O. V. Sanderfoot, J. D. Kaufman, B. Gardner

**Affiliations:** 1 School of Environmental and Forest Sciences, University of Washington, Seattle, WA USA; 2 Department of Environmental and Occupational Health Sciences, University of Washington, Seattle, WA USA

**Keywords:** Conservation biology, Ecological modelling, Urban ecology, Environmental impact

## Abstract

Birds living in developed areas contend with numerous stressors, including human disturbance and light, noise, and air pollution. COVID-19 pandemic lockdowns presented a unique opportunity to disentangle these effects during a period of reduced human activity. We launched a community science project in spring 2020 to explore drivers of site use by and detection of common birds in cities under lockdown in the U.S. Pacific Northwest. Our goals were twofold: (1) consider how intensity of urbanization, canopy cover, and availability of bird feeders and bird baths influenced avian habitat use; and (2) quantify how daily changes in weather, air pollution, and human mobility influenced detection of birds. We analyzed 6,640 surveys from 367 volunteers at 429 monitoring sites using occupancy models for 46 study species. Neither land cover nor canopy cover influenced site use by 50% of study species, suggesting that backyard birds may have used a wider range of habitats during lockdowns. Human mobility affected detection of 76% of study species, suggesting that birds exhibited species-specific behavioral responses to day-to-day changes in human activity beginning shortly after initial lockdown restrictions were implemented. Our study also showcases how existing community science platforms can be leveraged to support local monitoring efforts.

## Introduction

As urbanization intensifies globally^1^, it is increasingly important to understand the impacts of land use change and human activity on birds to mitigate potential adverse effects^2^ and avert further population declines^3^. Urbanization replaces natural vegetation with built structures and impervious surfaces, which leads to direct habitat loss for many species^2,4^. In addition, multiple environmental stressors known to affect avian health and behavior intensify along the urbanization gradient^4^, including the urban heat island effect^5–7^, artificial light at night^8–11^, anthropogenic noise^12–15^, air pollution^16,17^, and human disturbance^18,19^. Over time, these selective pressures have forced birds to avoid, adapt to, or exploit urban areas, ultimately influencing their distributions and community composition along the urban gradient^4,20^. However, quantifying the individual, collective, and interactive effects of urban stressors on birds is inherently challenging because these variables are often closely correlated^4,21^, making it difficult to tease apart how the built environment, pollution, and human disturbance affect bird behavior and species distributions.

In spring 2020, at the height of the first wave of the COVID-19 pandemic, more than half of the world’s human population was under lockdown in an effort to stem the tide of the evolving worldwide tragedy. These lockdowns, instituted to slow the spread of SARS-CoV-2, have presented a unique opportunity to study birds during a period of reduced human activity^22,23^, allowing researchers to begin to disentangle some of these effects. Vehicle traffic, air travel, and shipping activity plummeted^22^, resulting in a sudden, rapid decline in global human mobility (i.e., the movement of people)^24–27^. During this unprecedented moment in human history, now referred to as the “Anthropause”^23^, some cities under lockdown also observed temporary reductions in anthropogenic noise^28–30^ or air pollution^31–34^. The novel conditions of the Anthropause have allowed researchers to test hypotheses regarding the effects of specific types of human activity or environmental stressors on birds. For example, Hentati-Sundberg et al. found that with fewer tourists visiting a popular seabird colony in Sweden, white-tailed eagles (*Haliaeetus albicilla*) became more active, which indirectly led to reduced reproductive success of Common Murres (*Uria aalge*)^35^. Meanwhile, Derryberry et al. showed that male White-crowned Sparrows (*Zonotrichia leucophrys*) sang softer, lower-pitched songs when noise pollution declined during lockdowns in California’s Bay Area^36^. Recent studies have also demonstrated population-level impacts of lockdowns on avifauna. For example, Schrimpf et al. found that the abundance of 24 bird species changed in spring 2020 in counties and provinces in the United States and Canada where lockdown restrictions resulted in more extreme declines in human mobility^37^, and Estela et al. documented decreases in the species richness of nocturnal birds as lockdown restrictions eased and human mobility increased in Cali, Colombia^21^.

One common approach used in ecological studies to understand the distributions and habitat use of wildlife species are occupancy models^38^, which also provide a mechanism to examine impacts of reduced human mobility during COVID-19 lockdowns on bird behavior^39^. Occupancy models rely on repeated observations of a species at specific locations, allowing researchers to model where that species occurs on the landscape while accounting for imperfect detection (i.e., instances when observers fail to detect a species that is present). Typically, occupancy probability is the metric of interest, as this parameter provides information on species distributions. However, the detectability of a species reflects its availability and perceptibility—that is, whether a bird could be detected (i.e., it is visible or vocalizing) and whether it is noted by an observer^40^; therefore, shifts in detection probabilities can also potentially provide insight into the abundance or behavior of birds^39,41^. For example, time of day is often considered in the detection component of occupancy models for songbirds, as many species are more likely to be singing—and therefore detected—earlier in the morning. Environmental conditions may also influence detection of birds; for example, inhalation exposure to air pollution has been linked to adverse health impacts and behavioral changes in birds likely to impact their detectability^16^. Thus, occupancy models can provide a means for examining factors that drive the occurrence of species, but also for investigating factors that influence detection, which may relate to underlying changes in animal behavior.

With many long-term biodiversity monitoring programs on hiatus during COVID-19 pandemic lockdowns, researchers have recognized the value of community science data in studying birds during the Anthropause^37,42^, including in an occupancy modeling framework^39^. However, participation in community science also changed during the pandemic; some programs gained volunteers while others struggled to retain them^43–45^. In spring 2020, we launched a community science project using the eBird platform^46^ to monitor birds in cities under lockdown in the Pacific Northwest. Volunteers signed up to conduct weekly surveys of birds in their neighborhoods during a 3-month data collection campaign (April 1–June 30, 2020), which overlapped with shelter-in-place orders and school closures in the region. More than 900 people enrolled in the program; together, their observations constituted a large dataset of bird counts from repeated surveys at hundreds of locations that could be analyzed with occupancy models. Furthermore, by directly recruiting, training, and engaging volunteers, we were also able to establish a standardized sampling protocol and provide support for species identification, ensuring consistency and accuracy throughout data collection.

We combined this powerful community science dataset with data on multiple environmental variables to conduct an exploratory analysis of drivers of avian habitat use and detection of common, backyard birds in Pacific Northwest cities during the Anthropause. We had two main research objectives: First, we examined how the intensity of urbanization (characterized by land cover type) and canopy cover influenced avian habitat use. Second, we examined how daily changes in weather, air pollution, and human mobility influenced detection of birds. We also conducted a secondary analysis using a subset of surveys to consider how bird feeders and bird baths—resources often provided to birds by people in developed areas—influenced avian site use. We expected that drivers of site use by and detection of birds would vary by species, given the wide range of habitat preferences, life history strategies, and sensitivity to human activity represented by the 46 species included in our study (Appendix C). In addition, we hypothesized that human mobility would be inversely related to the probability of detecting songbirds (i.e., oscines)—a group of birds commonly detected by ear (that is, using audio cues)—due to concurrent reductions in background noise^36,47^.

Our study provides a snapshot of the distributions and detectability of backyard birds in cities under lockdown in the Pacific Northwest during the Anthropause. Although our analysis is exploratory, our findings offer valuable insight into which habitats and resources birds select for within highly heterogeneous developed areas and how day-to-day changes in weather, air pollution, and human mobility influence the detectability of birds—information that serves as a useful starting point in generating testable hypotheses to inform conservation policy and wildlife management^48,49^. The relationships we report in this paper are valuable because they describe avian responses to a collection of habitat features and urban stressors that are rarely considered simultaneously, providing a foundation for future studies and establishing a baseline for if and when human activity returns to pre-pandemic levels. By teasing out drivers of site use by and detection of birds in cities, we can learn more about species-specific sensitivity to human activity and tailor urban conservation strategies accordingly, helping birds thrive in an increasingly developed world.

## Methods

### Data collection

#### Data collection campaign

Volunteers signed up to monitor birds in their neighborhoods beginning April 1st, 2020. We enrolled volunteers on a rolling basis; at the end of our data collection campaign, we had received 917 responses to our online sign-up form. Participants selected their own monitoring sites, and we encouraged volunteers to survey birds in their yard or at a local green space they could safely access while following public health guidelines and without violating shelter-in-place orders. We asked volunteers to conduct 10-min, stationary point counts at their chosen site at least once a week, from April 1st through June 30th. They recorded all the birds they detected during a point count, using both visual and auditory cues, and submitted their observations from each survey as a complete checklist to eBird, an online database run by the Cornell Lab of Ornithology. eBird supports opportunistic data collection by birders who use a web application to submit checklists of species they observe and information about where and when observations took place (e.g., date, time)^46^. To help us identify checklists submitted as part of our data collection campaign in the eBird database, we asked volunteers to flag their checklists by adding the phrase “social distancing survey” to the trip comments field.

#### Volunteer questionnaire

In July 2020, we invited adult volunteers to participate in an online questionnaire to collect more information about their monitoring sites and their motivations for volunteering. This questionnaire allowed us to collect data on site characteristics that could not be assessed using publicly available datasets, including whether seed or suet feeders, hummingbird feeders, and/or bird baths were made available to birds at survey locations. We received responses from 284 volunteers. All methods were carried out in accordance with relevant guidelines and regulations, including guidelines for human subjects research. Our protocol and questionnaire were reviewed by the Human Subjects Division at the University of Washington (IRB ID: STUDY00010577) and determined to be exempt from a requirement for informed consent.

#### Bird observations

We downloaded all bird observations from checklists submitted in the U.S. states of Washington, Oregon, California, and Idaho and British Columbia, Canada from April 1–June 30, 2020 from the eBird database. To identify those checklists submitted by our volunteers, we first searched for some variation of the “social distancing survey” flag in the trip and/or species comments and then matched the observer IDs associated with those checklists to the names of our volunteers. We also used auxiliary information collected in the volunteer questionnaire to ascertain the IDs of any volunteers who did not flag their checklists in eBird. We were ultimately able to determine the observer IDs of 431 volunteers. Next, we filtered the checklists submitted by our volunteers using the *auk* package^50^ in R^51^. We only included checklists that met our survey protocol (i.e., complete, stationary surveys that were 9 to 11 min in duration).

**Figure 1:**
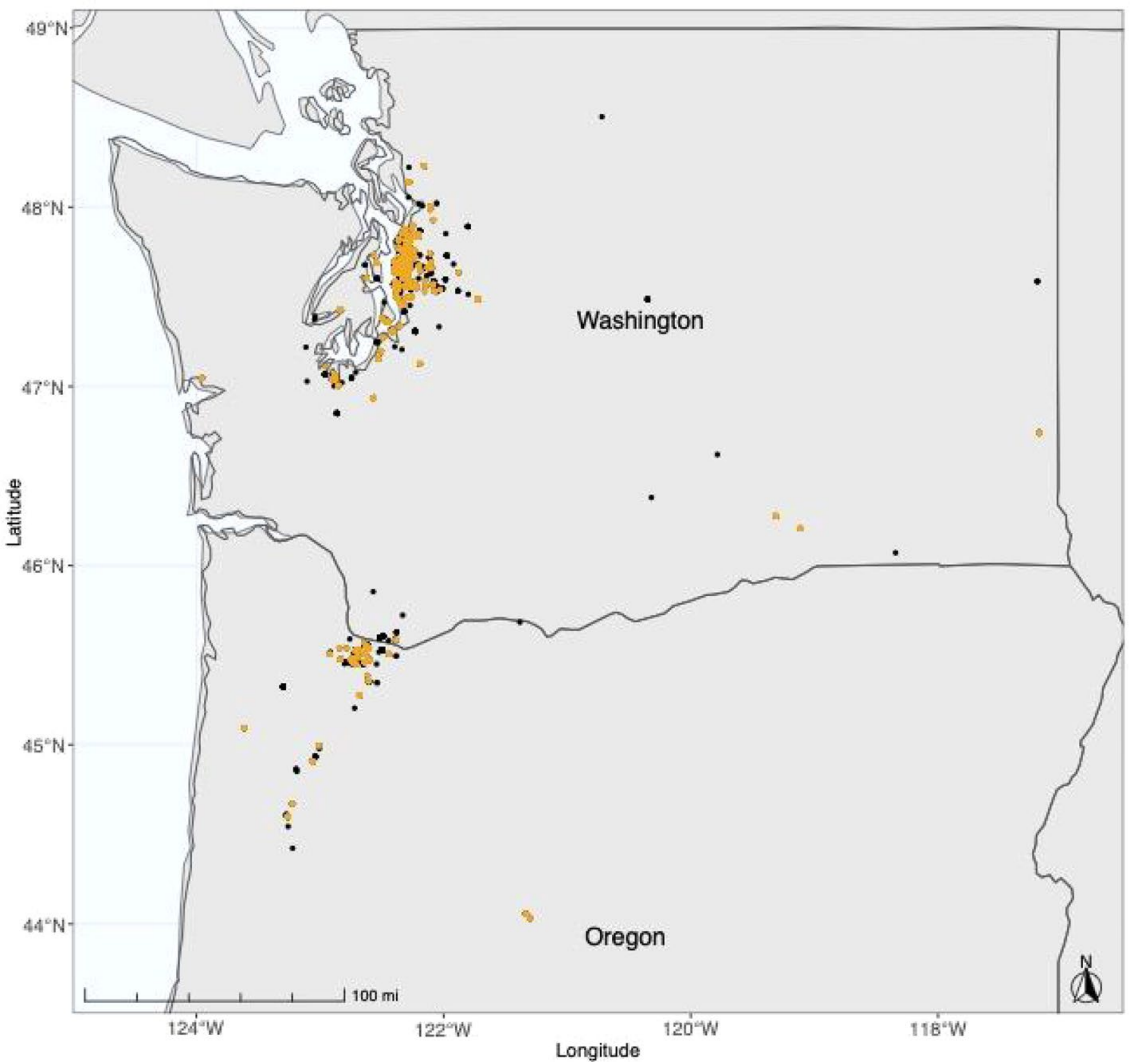
Map of study area, which includes the states of Washington and Oregon. Dots mark the locations of all monitoring sites included in our analysis. We had information on supplementary resources (i.e., availability of bird feeders and bird baths) for monitoring sites shown in orange. Map created using R^51^.

Finally, we binned checklists into circular, spatial units with a 100-m radius using a hierarchical clustering approach implemented using the *stats* package in R^51^. We considered each unit to be a unique site in our analysis. Therefore, all checklists assigned to a site represent surveys that took place within 100 m of the site’s center. Binning checklists ensured that all surveys conducted by volunteers at a single location were associated with the same site, regardless of inaccuracies in GPS locations measured by personal phones. This step also ensured that when volunteers happened to monitor nearby locations (e.g., neighboring yards), we considered their checklists to be surveys of the same site. Sites were heavily concentrated in major metropolitan areas in Washington and Oregon (e.g., Seattle, Portland); we therefore decided to focus our analysis on bird observations from just these two states (Fig. 1).

There were a total of 193 species observed by volunteers. We chose the species for this study based on the frequency and spatial distribution of detections. We focused on analyzing species that were observed at a minimum of 5% of monitoring sites on at least 150 sampling occasions to ensure that we had sufficient data to fit occupancy models. Forty-six species met these criteria, including both resident and migratory birds.

#### Environmental data

We used data from ground-based instruments included in the Environmental Protection Agency (EPA) Air Quality System (AQS)^52^ to characterize air pollution at monitoring sites, specifically the ambient concentration of fine particulate matter (PM_2.5_). PM_2.5_ includes all suspended solid and liquid particles smaller than 2.5 microns in aerodynamic diameter. Source apportionment studies show that human activity is an important driver of PM_2.5_ in urban areas, with vehicle traffic accounting for 24% of total PM_2.5_ in U.S. cities^53^. Due to the potentially complex relationships between urban air pollution and weather in cities^54^, we chose to consider only one pollutant in our analysis. We focused on PM_2.5_ because we felt more confident assessing temporal variation in this pollutant across our study area, as measurements were available from a greater number of instruments. We averaged the daily mean concentration of PM_2.5_ (μg/m^3^) across all sensors within Core-Based Statistical Areas (CBSAs; ; metro- or micropolitan statistical areas, or areas associated with at least one urban cluster with a minimum population of 10,000) for which a complete time series was available. This allowed us to capture temporal variation in PM_2.5_ within metropolitan and micropolitan areas. We determined the daily mean concentration of PM_2.5_ on the days surveys were conducted, based on the CBSA associated with each monitoring site. We excluded surveys from monitoring sites outside CBSAs, limiting our analysis to metro- and micropolitan areas.

To characterize shifts in human mobility during COVID-19 pandemic lockdowns, we relied on data from Google LLC’s Community Mobility Reports^55^, which have been widely used to study changes in human behavior during the COVID-19 pandemic^26,27^ and to examine effects of lockdowns on birds^21,37^. To measure human mobility, Google relies on data from its users’ mobile devices, specifically those who have agreed to share their Location History. The mobility reports provide a daily percent change in human mobility across various place categories from a baseline established in the weeks just prior to the pandemic, between January 3rd and February 6th, 2020. Data are available at the county scale. We used data on vehicle traffic from Waze, a navigation software company^56^, to compare traffic volume and human mobility in cities within our study area. We chose to use data on human mobility from the “retail and recreation” place category in our analysis because this time series was most highly correlated with that for traffic volume (R = 0.95).

We used data from the North American Regional Reanalysis (NARR) to capture variability in weather across surveys. We used the *ncdf4* package^57^ in R^51^ to extract daily mean air temperature and daily accumulated precipitation for each survey. NARR data were provided by the National Oceanic and Atmospheric Administration (NOAA) Physical Sciences Laboratory in Boulder, Colorado, USA from their website at https://psl.noaa.gov/.

To account for differences in habitat across monitoring sites, we extracted data on land cover and percent canopy cover from the 2016 National Land Cover Database (NLCD), developed by the Multi-Resolution Land Characteristics (MRLC) Consortium, in ArcMap^58,59^. Land cover classifications delineated by MRLC were grouped into three categories: highly developed (more than 50% impervious surface), less developed (less than 50% impervious surface), and natural areas (forest, shrubland, wetland, etc.). We obtained information on supplementary resources available to birds at monitoring sites from the online volunteer questionnaire (see above). We used responses from volunteers to build three binary, categorical variables to indicate availability of seed or suet feeders, hummingbird feeders, and/or bird baths at monitoring sites.

### Statistical analysis

We created survey-by-site detection histories for all 46 species included in the dataset. We analyzed these detection/non-detection data using single-season occupancy models, fit using the *unmarked* package^60^ in R ^51^. Occupancy models are commonly used by ecologists to explore bird distributions because they allow researchers to account for imperfect detection (i.e., the possibility of failing to detect a species, even though it is present). We defined the season as the duration of the data collection campaign (April 1–June 30, 2020), which overlaps with the breeding season for many study species (Appendix C).

In addition to the variables described above, we included a few additional covariates to account for how seasonal and daily variation in bird activity influenced detection of birds. We included day of year and day of year^2^ as predictors of detection, knowing that the probability of observing birds may vary seasonally and this temporal variation may be nonlinear. We also included an indicator variable for whether a survey was conducted on a Saturday or Sunday to account for differences in human activity on weekends. To adjust for the daily activity patterns of birds, we included time of day and time of day^2^ as covariates on detection. For each of the 46 study species, we modeled ${\psi }_{i}$, the probability that a species was present at monitoring site $i$, as:

$$logit\left({\psi }_{i}\right)= {\alpha }_{0} + {\alpha }_{1} x\,{land\,cover}_{i} + {{\alpha }_{2 }x\,canopy\,cover}_{i}$$

We modeled ${p}_{ij}$, the probability of detecting a species at site $i$ in survey $j$, as:

$$logit\left( {p_{{ij}} } \right) = \beta _{0} + \beta _{1} \;x\;day_{{ij}} + \beta _{2} \;x\;day_{{ij}} ^{2} + \beta _{3} \;x\;weekend_{{ij}} + \beta _{4} \;x\;time_{{ij}} + \beta _{5} \;x\;time_{{ij}} ^{2} + \beta _{6} \;x\;temperature_{{ij}} + \beta _{7} \;x\;precipitation_{{ij}} + \beta _{8} \;x\;mobility_{{ij}} + \beta _{9} \;x\;PM_{{2.5_{{ij}} }}$$

We computed 95% confidence intervals for all coefficients; we considered confidence intervals that did not overlap zero as indicative of statistically significant effects. All numeric variables were standardized prior to analysis to facilitate comparison of effect sizes.

The definition of a site is important in occupancy modeling, as it relates directly to inference regarding which areas are occupied by a species. However, determining if a site is “occupied” is difficult in contiguous habitat ^61^. For example, because the home ranges of our study species may exceed the size of monitoring sites, species may not be constantly present within a site or may be present at more than one site. Consequently, we interpreted the probability of occupancy ($\psi$) as the probability of use; that is, the probability that a species visited a monitoring site at least once during the study period. Covariates on $\psi$ therefore represent effects on site use, providing insight into what makes sites within urban areas attractive to birds. The probability of detecting birds ($p$) reflects both the probability that a species is present and *available* at a site during a survey and the probability that it is then *perceived* by an observer. Therefore, effects on $p$ may be due to shifts in avian abundance or behavior (e.g., movement, vocalization) that impact the visual and/or auditory cues relied on by observers or survey conditions that influence perceptibility (e.g., visibility, background noise) ^40^.

We also conducted a secondary analysis to investigate whether provision of supplementary resources influenced site use by birds. In this analysis, we considered the effects of bird feeders, hummingbird feeders, and bird baths on site use in addition to the variables included in the equations provided above. This secondary analysis was limited to a subset of surveys conducted at monitoring sites for which we had additional information on supplementary resources from the volunteer questionnaire.

Finally, we were concerned about the possibility of including multiple correlated predictors in the detection portion of our models; as such, we thoroughly explored the potential for multicollinearity. We calculated the Pearson’s correlation coefficient between all numeric variables included in the detection portion of our models; a correlation matrix is provided in Appendix A. We found |r|< 0.7 for all covariates. We also used the variance–covariance matrix of fitted model parameters to compute the Variance Inflation Factor (VIF) for all effects on detection; we chose a threshold of VIF > 5 as indicative of multicollinearity. Covariates in the detection model did not surpass the threshold for multicollinearity for all but three species: the VIF for the effects of day of year were high for Ruby-crowned Kinglets (*Regulus calendula*), Golden-crowned Sparrows (*Zonotrichia atricapilla*), and Yellow-rumped Warblers (*Setophaga coronata*). For these three species, we examined the impact of this multicollinearity by excluding day of year (both the linear and quadratic terms) from our models and comparing results. Results generally remained consistent, except that the effects of human mobility and temperature became statistically significant after day of year was removed for all three species. This was not unexpected because day of year was moderately correlated with these two covariates. In addition, after excluding day of year, the relationship between PM_2.5_ and detection of Golden-crowned Sparrows and the relationship between weekend and detection of Yellow-rumped Warblers became statistically significant. However, we chose to proceed with the full model because of the importance of accounting for seasonality when modeling detection probabilities and acknowledge that we may be underreporting the significance of human mobility and temperature for these three species.

## Results

Our final dataset included observations of 46 species from 6,640 eBird checklists submitted by 367 volunteers at 429 monitoring sites (Fig. 1). Our study included sites that captured a gradient of urbanization; of our 429 monitoring sites, 137 were in highly developed areas, 213 were in less developed areas, and 79 were in natural areas, and canopy cover ranged from 0 to 91%, with a median value of 21%.

**Figure 2:**
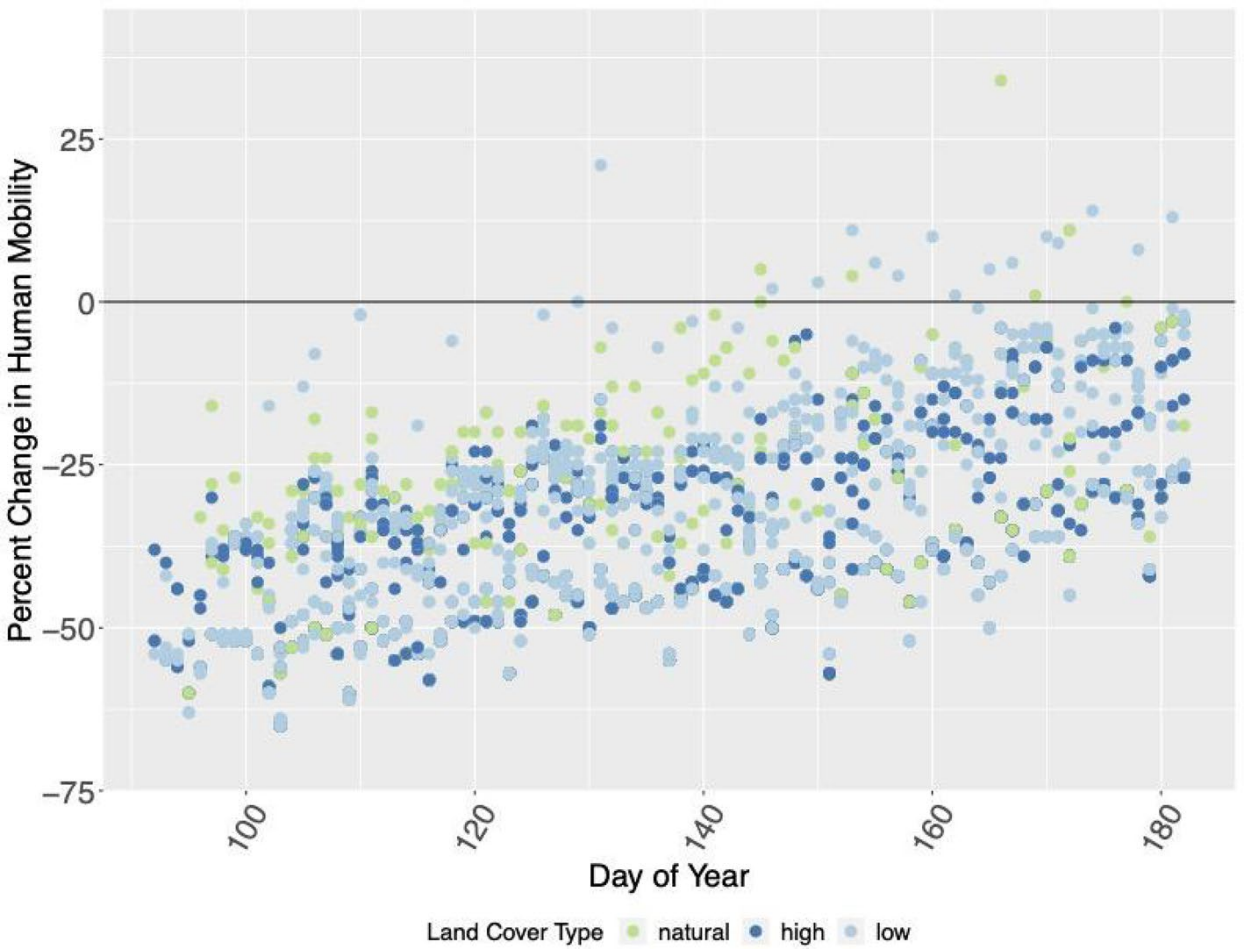
Daily percent change in human mobility relative to a pre-pandemic baseline over the course of the study period. Each dot represents a survey included in our analysis, color coded by the land cover type of the monitoring site (green = natural areas, dark blue = highly developed, light blue = less developed). Negative values indicate that human mobility was lower than it was before the pandemic. Note that human mobility was suppressed across the study area during COVID-19 pandemic lockdowns.

**Figure 3:**
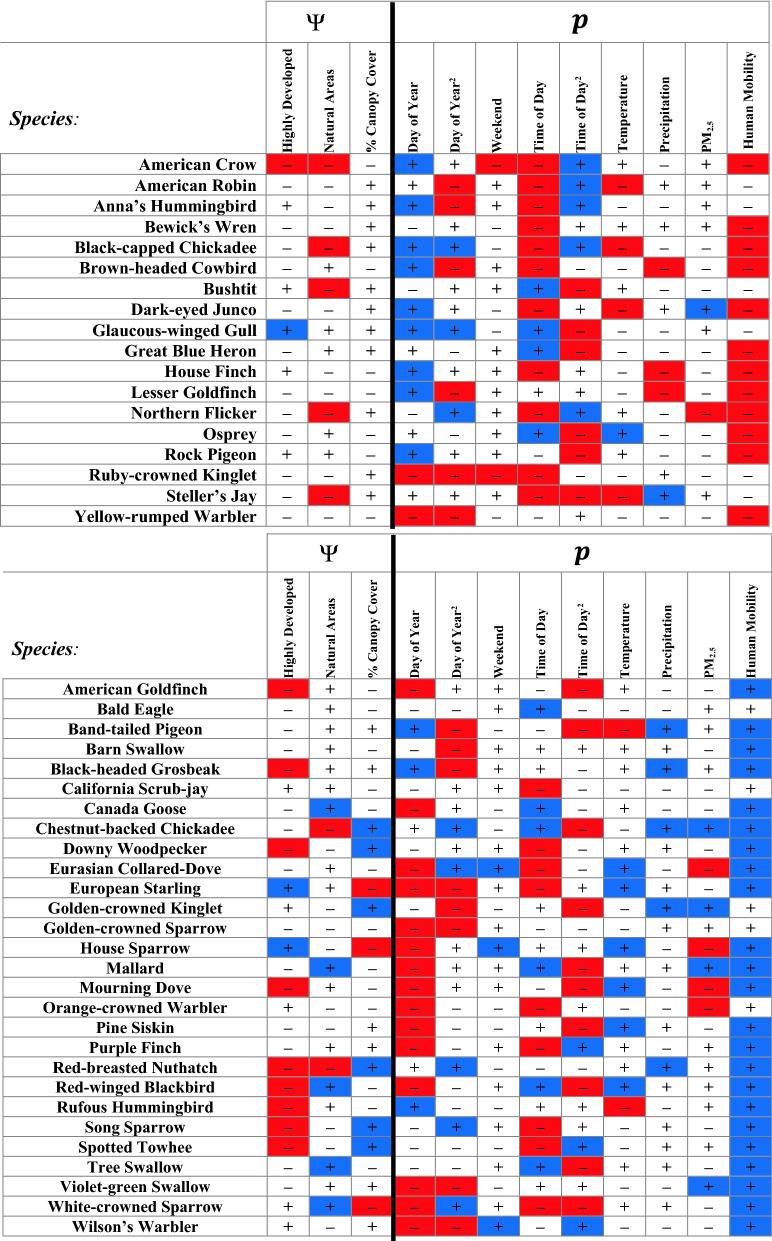
Results of single-season occupancy models by species. The top panel includes results for species that were less likely to be detected at higher levels of human mobility, and the bottom panel includes results for species that were more likely to be detected at higher levels of human mobility. The first three covariates were included as predictors of occupancy, with highly developed and natural areas shown relative to the less developed category (not shown). The next nine covariates (including day of year, day of year^2^, weekend, time of day, time of day^2^, temperature, precipitation, PM_2.5_, and human mobility) were included as predictors of detection. Blue boxes indicate statistically significant, positive effects (p < 0.05), and red boxes indicate statistically significant, negative effects. White boxes indicate effects with coefficient estimates that were not statistically different from zero. Complete model results are provided in Appendix B.

We found that both human mobility and air pollution affected the probability of detecting birds in cities during COVID-19 pandemic lockdowns in the U.S. Pacific Northwest. Human mobility gradually increased across the region during the data collection campaign as human behavior changed in the weeks following initial lockdown restrictions (Fig. 2). The daily percent change in human mobility had a statistically significant effect on the detection of 35 of the 46 study species (76%). As human mobility increased, 12 species were less likely to be detected and 23 species were more likely to be detected. PM_2.5_ had a statistically significant effect on detection of 10 of the 46 study species (22%) (Fig. 3).

We also found that land cover was a statistically significant predictor of site use for 22 of the 46 study species (48%). Canopy cover was a statistically significant predictor of site use for 9 study species (20%) (Fig. 3). As expected, seasonality, time of day, and weather affected the probability of detecting a wide range of species. Day of year and day of year squared had a statistically significant effect on the probability of detecting 29 and 22 species (63% and 48%), respectively. Weekends influenced the probability of detecting 5 species (11%). Time of day had a statistically significant effect on the detection of 65% of study species (30 total), and time of day^2^ had a statistically significant effect on detection of 52% of study species (24 total). Temperature influenced detection of 13 species (28%), and precipitation influenced detection of 9 species (20%) (Fig. 3). Complete model results are provided in Appendix B.

Our secondary analysis was limited to 5,459 surveys from 248 observers at 242 monitoring sites for which we had information on availability of bird feeders and bird baths. This analysis showed that provision of supplementary resources also influenced site use by our study species. Four species (9%) were more likely to be present at sites with seed or suet feeders, and 6 species (13%) were less likely to be present at sites with seed or suet feeders. Anna’s Hummingbird (*Calypte anna*) and Rufous Hummingbird (*Selasphorus rufus*) both showed strong, positive associations with the availability of hummingbird feeders. Finally, 6 of the 46 study species (13%) were more likely to use sites with bird baths; no species were negatively associated with this resource.

## Discussion

We found that after accounting for seasonality, time of day, weather, and air quality, daily shifts in human mobility affected the probability of detecting 76% of species included in our analysis (Fig. 3). This suggests that changes in human behavior following initial COVID-19 lockdown restrictions (Fig. 2) influenced detection of a wide range of common birds in the U.S. Pacific Northwest. Contrary to our expectations, we did not find consistent evidence that songbirds were more likely to be detected at lower levels of human mobility. We had expected that songbirds, often detected by ear (that is, by identifying songs and calls), would be more readily detected when human mobility was lower, due to corresponding declines in background noise. However, we found that human mobility was *positively* related to detection probability for half of our study species, including several songbirds. This suggests that at least some species changed their behavior to be either more perceptible (e.g., singing more) or more available (e.g., increased territoriality) when human mobility was higher in the surrounding area. While investigating the species-specific behavioral responses driving the relationships between human mobility and detection of birds was beyond the scope of our study, we considered several possible mechanisms that could explain our findings.

Changes in human mobility may have impacted the presence and availability of birds, presumably via changing the degree to which habitats experienced disturbance. Most of our monitoring sites were located in yards or public parks; the positive relationships we report between human mobility and detection probability could suggest that some birds altered their daily habitat use in response to increased human mobility, selecting for habitats like those monitored by our volunteers. If that is true, that could mean that these green spaces offer important refugia for birds in developed areas. Of the 23 species for which human mobility and detectability were significantly positively related, 14 (61%) were more likely to use less developed or natural areas or sites with greater canopy cover; on the other hand, none of the species with a significant negative relationship between human mobility and detectability were positively associated with natural areas. These findings indicate that the effects of human mobility on detection probability (whether positive or negative) are likely related to habitat selection and underlying occurrence patterns. However, because less developed areas may also have been less impacted by changes in human mobility as a result of lockdown restrictions (Fig. 2), we are limited in our ability to parse out the interaction between human mobility and habitat use. Furthermore, while Google’s Community Mobility Reports offer the best available data on human mobility^37^, data with a finer spatial resolution may be necessary to fully capture this relationship. Future studies should continue to investigate the relationship between human mobility and avian habitat use to assess how daily, seasonal, and extreme shifts in human mobility (as observed during the Anthropause) influence avian habitat use.

We also explored basic life history traits among the study species to determine potential biological reasons why one group of birds was more likely to be detected and the other less likely to be detected at higher levels of human mobility (Fig. 3). We considered habitat preferences, diet, foraging strategy, the timing of the breeding season, and nest type (Appendix C). We did not observe an obvious pattern, although we noted that species that breed later in the year were absent among those for which we observed a statistically significant, negative relationship between human mobility and detection. As birds are generally considered to be more active during the breeding season, this may indicate that species with later breeding seasons were less impacted by changes in human mobility because they still needed to be more active to breed even as human mobility increased over the course of our study. We included day of year as a linear and quadratic covariate on detection to account for seasonal variation in avian activity; nevertheless we expect that birds may have responded differently to daily shifts in human mobility based on their migratory and breeding status (Appendix C).

Relative abundance of species can also influence species-specific detection probabilities. For example, more individuals of a species present at a site will likely lead to increased detection of that species. Some species may have been more abundant near urban areas during COVID-19 pandemic lockdowns ^37^ . Migration may have also contributed to changes in local abundance of some species over the course of the study period. We did not explicitly account for abundance-induced heterogeneity in detection; thus, the relationships we report between human mobility and species-specific detection probabilities may also be due to changes in relative abundance.

We also explored how daily changes in weather and air pollution impacted detectability of birds. We found that temperature and precipitation were important predictors of detection in 28% and 20% of species, respectively. This is similar to the proportion of species (22%) for which detection was influenced by PM_2.5._ We expected that the lockdowns would reduce air pollution in our study area; however, PM_2.5_ concentrations increased in both Washington and Oregon in the spring of 2020 as compared to the previous five years ^32^. Regardless, air quality was quite good in our study area during the Anthropause—in our analysis, concentrations of PM_2.5_ ranged between 1 and 11.2 μg/m^3^, with a median value of 3.9 μg/m^3^, which was well below the 24-h National Ambient Air Quality Standard (NAAQS) of 35 μg/m^3^. This suggests that even at levels deemed safe for human health, air pollution may also be driving species-specific behavioral changes in birds that ultimately influence their detectability (e.g., movement, vocalization)^41,62^. Future studies should investigate how air and noise pollution from vehicle traffic jointly impact avian behavior and influence the detection and distributions of birds in cities^63^; regional variation in the environmental impacts of COVID-19 pandemic lockdowns may provide an ideal context in which to explore these relationships.

Given the diverse habitat preferences and dietary requirements of our study species (Appendix C), we expected that land cover type or percent canopy cover would be important predictors of site use for most study species. Surprisingly, we found that neither land cover nor canopy cover influenced site use by half of the species we considered in our analysis, which may suggest that reduced human activity during lockdowns allowed birds to use a wider range of habitats within cities. Our results also show that availability of bird feeders and bird baths influenced site use by birds; for example, we found that Anna’s Hummingbirds and Rufous Hummingbirds showed strong, positive associations with hummingbird feeders and six species (13%) were more likely to use sites with bird baths. Intriguingly, we did not observe a negative relationship between bird baths and site use for any species, which could indicate that bird baths only promote, never dissuade, visitation by birds. These results suggest that provision of supplementary resources influences avian community assemblages within developed areas^64^. However, site use by most study species was not influenced by availability of bird feeders or bird baths, whereas 50% of study species exhibited statistically significant relationships between site use and at least one other habitat covariate (land cover and/or canopy cover). This suggests that for most backyard birds, habitat is a stronger driver of where birds occur and spend time than provision of supplementary resources. Future studies should continue to explore if and how bird feeders and bird baths drive bird distributions^64,65^.

While our volunteers monitored birds at much larger spatial and temporal extents than we could have surveyed on our own, data collected by community scientists has caveats. In our study, volunteers selected the sites where they conducted their surveys. Without random site selection, our spatial sampling may not be representative, limiting inference to a larger population^66^. For example, if volunteers were generally from more affluent neighborhoods, they may have observed greater species richness at their monitoring sites (i.e., the luxury effect)^67^. In addition, volunteers with more birding experience may have selected monitoring sites with greater species diversity; however, in our study we believed most chose to survey birds near their homes. Data collected by volunteers may contain inconsistencies (e.g., species misidentifications) that would be less likely in data collected by professional scientists. We actively encouraged beginner birders to participate in this project, and 22% of questionnaire respondents indicated that they were beginner or novice birders. However, we found that while volunteers who described themselves as advanced birders were more likely to detect some study species, the effect of birding expertise on detection varied by species and within levels of experience—in fact, beginner birders were more likely than advanced birders to detect some species. Some community science volunteers may also be less likely than paid technicians to follow strict or detailed sampling protocols; we found that communicating frequently with volunteers to answer questions about data collection can improve data quality.

With cities on the rise around the world, it is imperative that we understand the collective impact of urban stressors on birds and identify opportunities to bolster the quality of habitats available to birds in developed areas. Our exploratory analysis provides insight into avian habitat use and bird behavior during a highly unusual period of low human activity, laying the groundwork for future studies to investigate specific mechanisms underlying relationships with land use, weather, air pollution, and human mobility. We also demonstrate that existing community science infrastructure can be leveraged to support rapid-response, regional monitoring programs in which project leads directly train and engage volunteers. Such programs could be a valuable tool in studying how birds respond to shifts in human activity, extreme weather, or other acute events. Volunteers may be particularly interested in participating in community science projects in times of crisis—85% of volunteers who responded to our online questionnaire indicated that the COVID-19 pandemic played a role in prompting them to sign up. We recommend greater use of community science in local monitoring projects and encourage researchers to consider the value of engaging directly with volunteers.

## Supplementary Information

Supplementary Information.

## Data Availability

Most of the data used in this analysis are publicly available (see links to databases provided in the methods section). However, our IRB does not allow us to share data collected in the online questionnaire filled out by volunteers.
