# Supplementary material for: Drivers of avian habitat use and detection of backyard birds in the Pacific Northwest during COVID-19 pandemic lockdowns
Source: Sci Rep. 2022 Aug 11;12:12655. doi: 10.1038/s41598-022-16406-w (PMC9372093; doi:10.1038/s41598-022-16406-w)
Supplement: Supplementary file 1 — Supplementary Information. [file 41598_2022_16406_MOESM1_ESM.docx]

**Appendix**

Appendix A: Correlation matrix

We were concerned that some of the effects on detection in our occupancy models may be correlated. We used a correlation matrix to examine the relationship between numeric effects on detection, which we have provided here. |r| values above .5 are bolded. As expected, there is a slight positive correlation between daily mean air temperature and day of year and between percent change in human mobility and day of year.

|  | Day of Year | Time | Temperature | Precipitation | Mobility | PM_2.5_ |
| --- | --- | --- | --- | --- | --- | --- |
| Day of Year | 1.000 | -0.029 | **0.662** | 0.062 | **0.573** | -0.430 |
| Time | -0.029 | 1.000 | -0.038 | 0.024 | -0.045 | 0.028 |
| Temperature | **0.662** | -0.038 | 1.000 | -0.184 | 0.373 | -0.078 |
| Precipitation | 0.062 | 0.024 | -0.184 | 1.000 | -0.094 | -0.328 |
| Mobility | **0.573** | -0.045 | 0.373 | -0.094 | 1.000 | -0.325 |
| PM_2.5_ | -0.430 | 0.028 | -0.078 | -0.328 | -0.325 | 1.000 |

Appendix B: Complete model results

The following tables present complete model results for each of the 46 study species included in our analysis. Estimates for intercepts and effects on both occupancy and detection are provided along with 95% confidence intervals. Effects that meet our threshold for statistical significance (p < 0.05) are bolded. The color coding indicates whether the information is relevant to the observation (blue) or state process (orange).

| **Species** | **American Crow** | **American Goldfinch** | **American Robin** | **Anna's Hummingbird** | **Bald Eagle** | **Band-tailed Pigeon** |
| --- | --- | --- | --- | --- | --- | --- |
| **Intercept (Ψ)** | 3.07 (2.31, 3.83) | -0.51 (-0.86, -0.15) | 2.34 (1.78, 2.9) | 1.04 (0.67, 1.4) | -0.52 (-0.96, -0.08) | -1.45 (-1.9, -1) |
| **High Developed** | **-1.39 (-2.38, -0.41)** | **-0.7 (-1.35, -0.05)** | -0.71 (-1.53, 0.12) | 0.31 (-0.35, 0.98) | -0.02 (-0.76, 0.72) | -0.69 (-1.63, 0.25) |
| **Natural Areas** | **-1.89 (-2.94, -0.84)** | 0.51 (-0.24, 1.27) | -0.41 (-1.47, 0.65) | -0.65 (-1.38, 0.08) | 0.66 (-0.31, 1.63) | 0.64 (-0.21, 1.5) |
| **Canopy Cover** | -0.09 (-0.51, 0.33) | -0.19 (-0.48, 0.11) | 0.31 (-0.19, 0.81) | 0.25 (-0.07, 0.57) | -0.14 (-0.5, 0.23) | 0.33 (-0.03, 0.69) |
| **Intercept (p)** | 0.35 (0.25, 0.46) | -1.53 (-1.73, -1.33) | -0.24 (-0.34, -0.14) | -0.38 (-0.48, -0.27) | -2.64 (-2.94, -2.34) | -1.07 (-1.37, -0.76) |
| **Day of Year** | **0.31 (0.22, 0.41)** | **-0.52 (-0.68, -0.36)** | 0.07 (-0.02, 0.16) | **0.22 (0.12, 0.32)** | -0.17 (-0.44, 0.1) | **0.48 (0.19, 0.77)** |
| **Day of Year^2^** | 0.04 (-0.01, 0.1) | 0.04 (-0.06, 0.14) | **-0.14 (-0.19, -0.08)** | **-0.09 (-0.15, -0.03)** | -0.07 (-0.24, 0.1) | **-0.51 (-0.71, -0.3)** |
| **Weekend** | **-0.22 (-0.35, -0.09)** | 0.15 (-0.08, 0.39) | 0.01 (-0.12, 0.13) | 0.01 (-0.13, 0.14) | 0.28 (-0.07, 0.63) | -0.2 (-0.6, 0.2) |
| **Time** | **-0.34 (-0.41, -0.28)** | -0.05 (-0.17, 0.07) | **-0.4 (-0.47, -0.34)** | **-0.18 (-0.24, -0.11)** | **0.34 (0.13, 0.55)** | -0.08 (-0.27, 0.12) |
| **Time^2^** | **0.15 (0.09, 0.21)** | **-0.15 (-0.27, -0.03)** | **0.45 (0.39, 0.52)** | **0.13 (0.07, 0.19)** | -0.06 (-0.24, 0.12) | **-0.35 (-0.57, -0.13)** |
| **Temperature** | 0.02 (-0.06, 0.1) | 0.11 (-0.02, 0.23) | **-0.1 (-0.18, -0.03)** | -0.08 (-0.16, 0) | -0.08 (-0.31, 0.15) | **-0.31 (-0.55, -0.08)** |
| **Precipitation** | -0.06 (-0.12, 0) | -0.04 (-0.15, 0.07) | 0.01 (-0.05, 0.07) | -0.07 (-0.13, 0) | -0.02 (-0.2, 0.15) | **0.15 (0.01, 0.3)** |
| **Mobility** | **-0.44 (-0.51, -0.37)** | **0.55 (0.44, 0.65)** | -0.01 (-0.07, 0.06) | -0.08 (-0.15, 0) | 0.16 (-0.03, 0.35) | **0.2 (0.02, 0.37)** |
| **PM_2.5_** | 0.05 (-0.02, 0.12) | -0.04 (-0.16, 0.08) | 0.06 (0, 0.13) | 0.01 (-0.06, 0.08) | 0.05 (-0.14, 0.23) | 0.21 (0, 0.43) |

| **Species** | **Barn Swallow** | **Bewick's Wren** | **Black-capped Chickadee** | **Black-headed Grosbeak** | **Brown-headed Cowbird** | **Bushtit** |
| --- | --- | --- | --- | --- | --- | --- |
| **Intercept (Ψ)** | -2.12 (-2.72, -1.52) | 0.16 (-0.16, 0.48) | 2.61 (1.94, 3.29) | -0.92 (-1.32, -0.53) | -1.47 (-1.94, -0.99) | 0.22 (-0.14, 0.59) |
| **High Developed** | -0.59 (-1.73, 0.54) | -0.06 (-0.62, 0.5) | -0.65 (-1.62, 0.31) | **-0.93 (-1.77, -0.09)** | -0.73 (-1.68, 0.22) | 0.66 (-0.06, 1.37) |
| **Natural Areas** | 0.89 (-0.11, 1.9) | -0.56 (-1.25, 0.12) | **-2.25 (-3.16, -1.35)** | 0.69 (-0.11, 1.49) | 0.63 (-0.28, 1.54) | **-0.92 (-1.74, -0.09)** |
| **Canopy Cover** | -0.26 (-0.74, 0.21) | 0.13 (-0.14, 0.4) | 0.25 (-0.17, 0.68) | 0.18 (-0.15, 0.51) | -0.01 (-0.39, 0.37) | 0.09 (-0.23, 0.41) |
| **Intercept (p)** | -2.02 (-2.5, -1.54) | -0.74 (-0.87, -0.6) | -0.11 (-0.21, -0.01) | -1.27 (-1.53, -1.01) | -1.81 (-2.2, -1.42) | -1.67 (-1.83, -1.5) |
| **Day of Year** | 0 (-0.39, 0.38) | -0.05 (-0.18, 0.08) | **0.26 (0.16, 0.35)** | **0.79 (0.51, 1.06)** | **1.01 (0.7, 1.33)** | -0.04 (-0.2, 0.12) |
| **Day of Year^2^** | **-0.36 (-0.6, -0.12)** | 0.02 (-0.05, 0.1) | **0.14 (0.08, 0.2)** | **-0.94 (-1.15, -0.73)** | **-0.25 (-0.46, -0.05)** | 0.02 (-0.07, 0.12) |
| **Weekend** | 0.35 (-0.15, 0.85) | -0.17 (-0.33, 0) | -0.12 (-0.24, 0) | 0.25 (-0.06, 0.57) | 0.16 (-0.23, 0.55) | 0.01 (-0.2, 0.21) |
| **Time** | 0.17 (-0.1, 0.45) | **-0.25 (-0.34, -0.17)** | **-0.27 (-0.33, -0.21)** | 0.03 (-0.13, 0.2) | **-0.67 (-0.95, -0.39)** | **0.23 (0.12, 0.34)** |
| **Time^2^** | 0 (-0.22, 0.23) | 0.04 (-0.04, 0.11) | **0.12 (0.06, 0.18)** | -0.06 (-0.21, 0.09) | -0.21 (-0.47, 0.04) | **-0.15 (-0.25, -0.05)** |
| **Temperature** | 0.09 (-0.24, 0.41) | 0.06 (-0.05, 0.16) | **-0.11 (-0.19, -0.03)** | 0.08 (-0.1, 0.26) | -0.06 (-0.28, 0.16) | 0.03 (-0.1, 0.17) |
| **Precipitation** | 0.14 (-0.07, 0.35) | 0.01 (-0.07, 0.09) | -0.01 (-0.07, 0.05) | **0.18 (0.05, 0.31)** | **-0.25 (-0.45, -0.04)** | -0.08 (-0.19, 0.03) |
| **Mobility** | **0.71 (0.42, 1.01)** | **-0.11 (-0.21, -0.01)** | **-0.28 (-0.34, -0.21)** | **0.5 (0.36, 0.64)** | **-0.22 (-0.43, -0.02)** | -0.01 (-0.14, 0.12) |
| **PM_2.5_** | -0.03 (-0.3, 0.24) | 0.09 (0, 0.17) | 0 (-0.07, 0.06) | 0.11 (-0.09, 0.3) | -0.16 (-0.39, 0.07) | -0.02 (-0.12, 0.09) |

| **Species** | **California Scrub-jay** | **Canada Goose** | **Chestnut-backed Chickadee** | **Dark-eyed Junco** | **Downy Woodpecker** | **Eurasian Collared-Dove** |
| --- | --- | --- | --- | --- | --- | --- |
| **Intercept (Ψ)** | -1.43 (-1.86, -1.01) | -0.94 (-1.38, -0.51) | -0.15 (-0.48, 0.18) | 1.98 (1.45, 2.51) | -0.63 (-1.05, -0.2) | -1.66 (-2.23, -1.08) |
| **High Developed** | 0.55 (-0.11, 1.21) | -0.47 (-1.24, 0.29) | -0.33 (-0.92, 0.25) | -0.34 (-1.13, 0.45) | **-1.03 (-1.88, -0.17)** | -0.16 (-1.14, 0.83) |
| **Natural Areas** | 0.17 (-0.75, 1.09) | **1.46 (0.55, 2.36)** | **-1 (-1.76, -0.24)** | -0.71 (-1.69, 0.27) | -0.22 (-1.16, 0.71) | 0.05 (-1.09, 1.18) |
| **Canopy Cover** | -0.36 (-0.74, 0.01) | -0.32 (-0.69, 0.04) | **0.37 (0.08, 0.66)** | 0.3 (-0.13, 0.72) | **0.43 (0.06, 0.81)** | -0.27 (-0.77, 0.22) |
| **Intercept (p)** | -1.79 (-2.1, -1.47) | -2.43 (-2.75, -2.11) | -1.06 (-1.22, -0.89) | 0.03 (-0.07, 0.12) | -2.52 (-2.87, -2.18) | -3.2 (-3.77, -2.64) |
| **Day of Year** | -0.1 (-0.33, 0.12) | **-0.72 (-0.98, -0.45)** | 0.03 (-0.11, 0.17) | **0.35 (0.26, 0.44)** | -0.08 (-0.36, 0.21) | **-1.54 (-1.89, -1.2)** |
| **Day of Year^2^** | 0.14 (0, 0.28) | 0.01 (-0.16, 0.18) | **0.15 (0.06, 0.23)** | 0.01 (-0.04, 0.07) | 0.16 (-0.01, 0.34) | **0.3 (0.11, 0.49)** |
| **Weekend** | 0.24 (-0.08, 0.56) | -0.02 (-0.38, 0.34) | -0.01 (-0.21, 0.18) | -0.08 (-0.2, 0.04) | 0.16 (-0.23, 0.55) | **0.51 (0.08, 0.95)** |
| **Time** | **-0.31 (-0.48, -0.15)** | **0.27 (0.07, 0.46)** | **0.13 (0.03, 0.22)** | **-0.22 (-0.28, -0.15)** | **-0.34 (-0.56, -0.11)** | **-0.37 (-0.69, -0.05)** |
| **Time^2^** | -0.1 (-0.28, 0.09) | -0.09 (-0.25, 0.08) | **-0.15 (-0.25, -0.06)** | 0.05 (-0.01, 0.11) | -0.01 (-0.21, 0.19) | -0.15 (-0.46, 0.17) |
| **Temperature** | -0.1 (-0.28, 0.09) | 0.09 (-0.14, 0.31) | -0.02 (-0.14, 0.1) | **-0.21 (-0.29, -0.14)** | 0.06 (-0.18, 0.29) | **0.7 (0.5, 0.9)** |
| **Precipitation** | -0.12 (-0.3, 0.05) | -0.11 (-0.3, 0.08) | **0.11 (0.03, 0.2)** | 0.01 (-0.04, 0.07) | 0.09 (-0.09, 0.26) | 0 (-0.23, 0.22) |
| **Mobility** | 0.04 (-0.12, 0.21) | **0.33 (0.17, 0.49)** | **0.37 (0.27, 0.46)** | **-0.15 (-0.22, -0.09)** | **0.24 (0.06, 0.42)** | **1.14 (0.86, 1.42)** |
| **PM_2.5_** | -0.02 (-0.17, 0.14) | -0.02 (-0.19, 0.15) | **0.12 (0.02, 0.22)** | **0.1 (0.04, 0.17)** | -0.08 (-0.29, 0.13) | -0.45 (-0.67, -0.23) |

| **Species** | **European Starling** | **Glaucous-winged Gull** | **Golden-crowned Kinglet** | **Golden-crowned Sparrow** | **Great Blue Heron** | **House Finch** |
| --- | --- | --- | --- | --- | --- | --- |
| **Intercept (Ψ)** | -0.13 (-0.45, 0.2) | -2.09 (-2.59, -1.59) | -1.43 (-1.89, -0.97) | -0.58 (-0.98, -0.18) | -1.4 (-1.85, -0.94) | 0.83 (0.47, 1.18) |
| **High Developed** | **0.65 (0.05, 1.26)** | **0.78 (0.02, 1.55)** | 0.45 (-0.32, 1.23) | -0.56 (-1.25, 0.13) | -0.47 (-1.33, 0.38) | 0.47 (-0.22, 1.15) |
| **Natural Areas** | 0.54 (-0.18, 1.25) | 0.61 (-0.3, 1.53) | -0.56 (-1.63, 0.51) | -0.57 (-1.48, 0.35) | 0.75 (-0.18, 1.67) | -0.57 (-1.25, 0.1) |
| **Canopy Cover** | **-0.3 (-0.58, -0.03)** | 0.1 (-0.25, 0.46) | **0.55 (0.18, 0.92)** | -0.28 (-0.63, 0.07) | 0.04 (-0.33, 0.42) | -0.28 (-0.56, 0) |
| **Intercept (p)** | -1.04 (-1.18, -0.89) | -1.28 (-1.54, -1.02) | -1.52 (-1.84, -1.21) | -4.4 (-5.12, -3.68) | -1.52 (-1.88, -1.17) | -0.32 (-0.43, -0.21) |
| **Day of Year** | **-0.19 (-0.31, -0.07)** | **0.34 (0.06, 0.61)** | -0.11 (-0.4, 0.19) | **-5.9 (-7.31, -4.5)** | 0.26 (-0.04, 0.56) | **0.22 (0.12, 0.31)** |
| **Day of Year^2^** | **-0.1 (-0.18, -0.03)** | **0.16 (0.02, 0.31)** | **-0.23 (-0.41, -0.05)** | **-2.31 (-3, -1.61)** | -0.05 (-0.24, 0.15) | 0.05 (-0.01, 0.11) |
| **Weekend** | 0.14 (-0.03, 0.31) | -0.1 (-0.43, 0.23) | -0.17 (-0.56, 0.22) | 0.15 (-0.19, 0.49) | 0.18 (-0.24, 0.6) | 0.06 (-0.07, 0.2) |
| **Time** | **-0.24 (-0.32, -0.15)** | **0.52 (0.33, 0.72)** | 0.13 (-0.06, 0.32) | -0.1 (-0.3, 0.1) | **0.93 (0.67, 1.19)** | **-0.25 (-0.32, -0.19)** |
| **Time^2^** | 0.01 (-0.07, 0.1) | **-0.19 (-0.35, -0.04)** | **-0.3 (-0.51, -0.1)** | -0.16 (-0.35, 0.04) | **-0.77 (-0.99, -0.56)** | 0.02 (-0.05, 0.08) |
| **Temperature** | **0.13 (0.04, 0.23)** | -0.14 (-0.36, 0.08) | -0.04 (-0.28, 0.2) | -0.17 (-0.41, 0.08) | -0.01 (-0.25, 0.24) | -0.03 (-0.1, 0.05) |
| **Precipitation** | 0.03 (-0.05, 0.12) | 0 (-0.19, 0.18) | **0.21 (0.06, 0.37)** | 0.03 (-0.14, 0.21) | -0.04 (-0.26, 0.18) | **-0.08 (-0.15, -0.02)** |
| **Mobility** | **0.28 (0.19, 0.37)** | -0.18 (-0.43, 0.07) | 0.16 (-0.08, 0.4) | 0.12 (-0.06, 0.3) | **-0.29 (-0.54, -0.04)** | **-0.13 (-0.2, -0.05)** |
| **PM_2.5_** | -0.01 (-0.11, 0.08) | 0.13 (-0.03, 0.3) | **0.21 (0.02, 0.39)** | 0.05 (-0.09, 0.2) | -0.14 (-0.38, 0.1) | -0.05 (-0.12, 0.02) |

| **Species** | **House Sparrow** | **Lesser Goldfinch** | **Mallard** | **Mourning Dove** | **Northern Flicker** | **Orange-crowned Warbler** |
| --- | --- | --- | --- | --- | --- | --- |
| **Intercept (Ψ)** | -0.69 (-1.02, -0.36) | -2.34 (-2.86, -1.82) | -1.27 (-1.66, -0.88) | -1.22 (-1.66, -0.77) | 1.42 (0.95, 1.89) | -0.79 (-1.22, -0.36) |
| **High Developed** | **0.65 (0.09, 1.21)** | -0.92 (-2.01, 0.18) | -0.37 (-1.09, 0.35) | **-1.18 (-2.12, -0.25)** | -0.34 (-1.07, 0.39) | 0.01 (-0.71, 0.74) |
| **Natural Areas** | -0.27 (-0.99, 0.44) | -0.47 (-1.75, 0.81) | **1.15 (0.42, 1.87)** | 0.68 (-0.18, 1.54) | **-0.94 (-1.81, -0.07)** | -0.2 (-1.15, 0.74) |
| **Canopy Cover** | **-0.32 (-0.61, -0.03)** | -0.25 (-0.76, 0.26) | -0.05 (-0.35, 0.26) | -0.22 (-0.59, 0.16) | 0.21 (-0.18, 0.61) | -0.14 (-0.51, 0.23) |
| **Intercept (p)** | -0.98 (-1.14, -0.82) | 0.1 (-0.32, 0.51) | -1.43 (-1.73, -1.14) | -2.13 (-2.58, -1.68) | -1.65 (-1.78, -1.51) | -2.61 (-2.96, -2.27) |
| **Day of Year** | **-0.41 (-0.54, -0.27)** | **0.45 (0.18, 0.72)** | **-0.45 (-0.69, -0.21)** | **-0.98 (-1.23, -0.74)** | -0.02 (-0.14, 0.09) | **-0.7 (-0.99, -0.41)** |
| **Day of Year^2^** | 0.04 (-0.05, 0.12) | **-0.23 (-0.41, -0.06)** | 0.02 (-0.13, 0.17) | 0.12 (-0.03, 0.28) | **0.28 (0.21, 0.35)** | -0.11 (-0.3, 0.08) |
| **Weekend** | **0.25 (0.06, 0.45)** | 0.04 (-0.34, 0.43) | 0.12 (-0.21, 0.44) | 0.3 (-0.06, 0.66) | 0.04 (-0.11, 0.19) | -0.02 (-0.42, 0.37) |
| **Time** | 0.02 (-0.08, 0.12) | 0.05 (-0.15, 0.25) | **0.44 (0.24, 0.64)** | -0.17 (-0.38, 0.05) | **-0.24 (-0.32, -0.16)** | **-0.58 (-0.8, -0.37)** |
| **Time^2^** | 0.04 (-0.05, 0.14) | 0.11 (-0.11, 0.32) | **-0.32 (-0.48, -0.15)** | **-0.25 (-0.45, -0.05)** | **0.12 (0.05, 0.2)** | 0.19 (0, 0.38) |
| **Temperature** | **0.13 (0.02, 0.24)** | -0.06 (-0.26, 0.14) | 0.04 (-0.17, 0.24) | **0.61 (0.44, 0.78)** | 0.02 (-0.08, 0.12) | -0.01 (-0.26, 0.24) |
| **Precipitation** | -0.09 (-0.19, 0.01) | **-0.3 (-0.5, -0.11)** | 0.11 (-0.04, 0.27) | -0.02 (-0.2, 0.17) | -0.06 (-0.13, 0.02) | -0.15 (-0.37, 0.06) |
| **Mobility** | **0.42 (0.3, 0.54)** | **-0.56 (-0.78, -0.34)** | **0.36 (0.19, 0.54)** | **0.64 (0.46, 0.81)** | **-0.3 (-0.39, -0.21)** | 0.15 (-0.06, 0.35) |
| **PM_2.5_** | **-0.12 (-0.22, -0.02)** | -0.1 (-0.3, 0.1) | **0.18 (0.03, 0.34)** | **-0.26 (-0.42, -0.09)** | **-0.1 (-0.17, -0.02)** | **-0.32 (-0.51, -0.12)** |

| **Species** | **Osprey** | **Pine Siskin** | **Purple Finch** | **Red-breasted Nuthatch** | **Red-winged Blackbird** | **Rock Pigeon** |
| --- | --- | --- | --- | --- | --- | --- |
| **Intercept (Ψ)** | -1.7 (-2.19, -1.21) | -1.28 (-1.66, -0.91) | -1.88 (-2.38, -1.39) | -0.17 (-0.49, 0.16) | -1.7 (-2.11, -1.28) | -1.3 (-1.72, -0.88) |
| **High Developed** | -0.36 (-1.22, 0.5) | -0.74 (-1.51, 0.03) | -0.11 (-1.01, 0.79) | **-0.6 (-1.19, -0.01)** | **-0.93 (-1.8, -0.06)** | 0.27 (-0.43, 0.97) |
| **Natural Areas** | 0.83 (-0.14, 1.8) | -0.3 (-1.11, 0.51) | 0.62 (-0.28, 1.51) | **-0.74 (-1.47, -0.01)** | **0.94 (0.21, 1.68)** | 0.29 (-0.62, 1.2) |
| **Canopy Cover** | -0.35 (-0.78, 0.09) | 0.17 (-0.14, 0.48) | 0.21 (-0.16, 0.57) | **0.4 (0.13, 0.68)** | -0.16 (-0.49, 0.17) | -0.25 (-0.61, 0.11) |
| **Intercept (p)** | -1.26 (-1.59, -0.92) | -0.65 (-0.87, -0.43) | -1.97 (-2.33, -1.61) | -0.99 (-1.15, -0.84) | -0.34 (-0.62, -0.06) | -1.7 (-1.96, -1.44) |
| **Day of Year** | 0.09 (-0.24, 0.42) | **-0.62 (-0.81, -0.43)** | **-0.51 (-0.79, -0.24)** | 0.02 (-0.11, 0.16) | **-0.66 (-0.86, -0.46)** | **0.4 (0.18, 0.62)** |
| **Day of Year^2^** | -0.06 (-0.26, 0.15) | -0.12 (-0.24, 0) | -0.02 (-0.19, 0.15) | **0.11 (0.03, 0.19)** | -0.04 (-0.17, 0.1) | 0.03 (-0.1, 0.17) |
| **Weekend** | 0.19 (-0.24, 0.62) | -0.11 (-0.36, 0.15) | 0.34 (-0.04, 0.72) | -0.07 (-0.25, 0.11) | 0.23 (-0.08, 0.54) | 0.12 (-0.19, 0.43) |
| **Time** | **1.41 (1.04, 1.78)** | 0.13 (0, 0.25) | **-0.37 (-0.57, -0.16)** | -0.07 (-0.16, 0.02) | **0.55 (0.39, 0.72)** | -0.07 (-0.24, 0.09) |
| **Time^2^** | **-1.02 (-1.28, -0.77)** | **-0.17 (-0.29, -0.05)** | **0.2 (0.03, 0.38)** | -0.02 (-0.11, 0.07) | **-0.54 (-0.7, -0.37)** | **-0.19 (-0.35, -0.04)** |
| **Temperature** | **0.47 (0.18, 0.77)** | **0.29 (0.14, 0.45)** | 0.01 (-0.21, 0.24) | 0 (-0.11, 0.11) | **0.34 (0.18, 0.5)** | 0.06 (-0.11, 0.23) |
| **Precipitation** | -0.01 (-0.24, 0.21) | 0.03 (-0.08, 0.14) | -0.02 (-0.2, 0.15) | **0.1 (0.02, 0.18)** | 0.1 (-0.05, 0.25) | -0.14 (-0.31, 0.03) |
| **Mobility** | **-0.38 (-0.62, -0.14)** | **0.4 (0.28, 0.52)** | **0.62 (0.44, 0.79)** | **0.2 (0.11, 0.29)** | **0.32 (0.18, 0.46)** | **-0.32 (-0.53, -0.12)** |
| **PM_2.5_** | -0.22 (-0.48, 0.04) | -0.01 (-0.13, 0.12) | 0.05 (-0.13, 0.23) | 0.02 (-0.07, 0.12) | 0.04 (-0.11, 0.19) | -0.03 (-0.2, 0.13) |

| **Species** | **Ruby-crowned Kinglet** | **Rufous Hummingbird** | **Song Sparrow** | **Spotted Towhee** | **Steller's Jay** | **Tree Swallow** |
| --- | --- | --- | --- | --- | --- | --- |
| **Intercept (Ψ)** | -0.79 (-1.26, -0.31) | -0.66 (-1.08, -0.25) | 0.66 (0.32, 0.99) | 0.66 (0.32, 1) | 1.16 (0.74, 1.58) | -1.48 (-1.96, -1) |
| **High Developed** | -0.48 (-1.31, 0.35) | **-0.96 (-1.77, -0.16)** | **-0.57 (-1.12, -0.02)** | **-0.6 (-1.17, -0.04)** | -0.29 (-0.98, 0.41) | -0.06 (-0.88, 0.75) |
| **Natural Areas** | -0.04 (-1.13, 1.05) | 0.65 (-0.19, 1.5) | -0.6 (-1.26, 0.07) | -0.37 (-1.07, 0.33) | **-0.98 (-1.77, -0.18)** | **1.02 (0.13, 1.92)** |
| **Canopy Cover** | 0.12 (-0.28, 0.52) | -0.23 (-0.57, 0.12) | **0.33 (0.05, 0.6)** | **0.39 (0.09, 0.69)** | 0.26 (-0.09, 0.62) | -0.3 (-0.7, 0.09) |
| **Intercept (p)** | -7.28 (-9.03, -5.53) | -2.17 (-2.47, -1.86) | -0.58 (-0.7, -0.46) | -0.54 (-0.67, -0.42) | -1.33 (-1.47, -1.19) | -1.95 (-2.28, -1.61) |
| **Day of Year** | **-8.24 (-11.27, -5.22)** | **0.26 (0.01, 0.51)** | -0.06 (-0.17, 0.04) | -0.1 (-0.21, 0.01) | 0.1 (-0.03, 0.22) | -0.24 (-0.52, 0.03) |
| **Day of Year^2^** | **-2.65 (-3.97, -1.34)** | -0.15 (-0.32, 0.01) | **0.1 (0.03, 0.16)** | -0.07 (-0.14, 0) | 0.07 (-0.01, 0.14) | -0.05 (-0.23, 0.13) |
| **Weekend** | **-0.66 (-1.16, -0.15)** | -0.08 (-0.44, 0.29) | 0.07 (-0.08, 0.22) | -0.04 (-0.19, 0.11) | 0.04 (-0.13, 0.2) | 0 (-0.4, 0.41) |
| **Time** | **-0.4 (-0.66, -0.14)** | 0.03 (-0.16, 0.22) | **-0.23 (-0.3, -0.15)** | **-0.3 (-0.38, -0.23)** | **-0.2 (-0.29, -0.12)** | **0.26 (0.03, 0.49)** |
| **Time^2^** | -0.08 (-0.34, 0.19) | 0.02 (-0.15, 0.19) | 0.05 (-0.02, 0.11) | **0.1 (0.03, 0.17)** | **-0.09 (-0.18, -0.01)** | **-0.29 (-0.49, -0.1)** |
| **Temperature** | 0 (-0.38, 0.38) | **-0.24 (-0.44, -0.03)** | -0.04 (-0.13, 0.05) | -0.08 (-0.17, 0.02) | **-0.16 (-0.27, -0.06)** | 0.17 (-0.05, 0.39) |
| **Precipitation** | 0.05 (-0.19, 0.3) | 0 (-0.17, 0.17) | 0.06 (-0.01, 0.13) | 0.02 (-0.05, 0.09) | **0.08 (0.01, 0.16)** | 0.01 (-0.19, 0.21) |
| **Mobility** | -0.39 (-0.78, 0) | **0.38 (0.23, 0.53)** | **0.11 (0.04, 0.18)** | **0.33 (0.25, 0.4)** | 0 (-0.09, 0.09) | **0.78 (0.59, 0.97)** |
| **PM_2.5_** | -0.04 (-0.23, 0.15) | 0.15 (-0.03, 0.34) | -0.05 (-0.12, 0.02) | 0.01 (-0.07, 0.08) | 0.03 (-0.06, 0.11) | -0.05 (-0.26, 0.15) |

| **Species** | **Violet-green Swallow** | **White-crowned Sparrow** | **Wilson's Warbler** | **Yellow-rumped Warbler** |
| --- | --- | --- | --- | --- |
| **Intercept (Ψ)** | -1.31 (-1.71, -0.92) | -0.88 (-1.24, -0.51) | -0.67 (-1.08, -0.26) | -0.52 (-0.95, -0.1) |
| **High Developed** | -0.44 (-1.19, 0.31) | 0.1 (-0.51, 0.71) | 0.44 (-0.27, 1.14) | -0.3 (-1.01, 0.4) |
| **Natural Areas** | 0.45 (-0.3, 1.2) | **0.79 (0.04, 1.54)** | -0.45 (-1.37, 0.46) | -0.04 (-0.97, 0.9) |
| **Canopy Cover** | 0.1 (-0.21, 0.41) | **-0.4 (-0.71, -0.08)** | 0.27 (-0.07, 0.61) | -0.22 (-0.57, 0.13) |
| **Detection (p)** | -1.11 (-1.37, -0.86) | -1.4 (-1.61, -1.19) | -1.88 (-2.16, -1.6) | -4.36 (-5.07, -3.65) |
| **Day of Year** | **-0.37 (-0.58, -0.16)** | **-0.51 (-0.67, -0.35)** | **-0.62 (-0.89, -0.34)** | **-6.25 (-7.77, -4.73)** |
| **Day of Year^2^** | **-0.29 (-0.43, -0.15)** | **0.17 (0.06, 0.28)** | **-1.03 (-1.27, -0.8)** | **-3.48 (-4.33, -2.63)** |
| **Weekend** | -0.18 (-0.49, 0.14) | 0.09 (-0.14, 0.33) | **0.4 (0.07, 0.74)** | -0.37 (-0.76, 0.01) |
| **Time** | 0.07 (-0.08, 0.22) | **-0.24 (-0.37, -0.1)** | -0.09 (-0.25, 0.08) | -0.03 (-0.24, 0.19) |
| **Time^2^** | 0.04 (-0.09, 0.18) | **-0.26 (-0.39, -0.14)** | **0.19 (0.04, 0.34)** | 0.17 (-0.02, 0.36) |
| **Temperature** | -0.1 (-0.29, 0.08) | 0.05 (-0.08, 0.18) | -0.19 (-0.4, 0.03) | -0.22 (-0.52, 0.08) |
| **Precipitation** | -0.11 (-0.26, 0.04) | 0.07 (-0.05, 0.19) | -0.16 (-0.33, 0.01) | -0.14 (-0.34, 0.05) |
| **Mobility** | **0.77 (0.64, 0.91)** | **0.3 (0.19, 0.41)** | **0.62 (0.42, 0.83)** | **-0.5 (-0.76, -0.25)** |
| **PM_2.5_** | **0.22 (0.06, 0.37)** | -0.06 (-0.18, 0.05) | -0.09 (-0.28, 0.11) | -0.06 (-0.25, 0.12) |

Appendix C: Life histories of study species

Our study species exhibited a wide range of life history strategies. Here we provide a brief overview of the ecology of each species, in alphabetical order. We focus on migratory status, the timing of the breeding season, nest type, preferred habitat, diet, and behavior. To characterize the life histories of our study species, we relied on resources available from All About Birds, Birds of the World, and local field guides.

| **Species** | **Status** | **Breeding starts…** | **Breeding ends…** | **Nest type** | **Habitat** | **Diet** | **Behavior** |
| --- | --- | --- | --- | --- | --- | --- | --- |
| American Crow | resident | early Apr | early Jun | Trees | Open Woodlands | Ominvore | Ground Forager |
| American Goldfinch | resident | late May | late Aug | Shrubs | Open Woodlands | Granivore | Foliage Gleaner |
| American Robin | resident | early March | late Jul | Trees | Open Woodlands | Insectivore | Ground Forager |
| Anna's Hummingbird | resident | mid-Feb | late May | Trees | Open Woodlands | Nectarivore | Hovering |
| Bald Eagle | resident (western WA and OR); winter (eastern WA and OR) | mid-Mar | mid-Jul | Trees | Forests | Piscivore | Soaring |
| Band-tailed Pigeon | summer | late Apr | mid-Oct | Trees | Forests | Granivore | Foliage Gleaner |
| Barn Swallow | summer | early Jun | mid-Aug | Buildings | Grasslands | Insectivore | Aerial Forager |
| Bewick's Wren | resident | mid-Feb | early Aug | Cavities | Open Woodlands | Insectivore | Foliage Gleaner |
| Black-capped Chickadee | resident | early Apr | early Aug | Cavities | Forests | Insectivore | Foliage Gleaner |
| Black-headed Grosbeak | summer | early Jun | late Jul | Trees | Forests | Insectivore | Foliage Gleaner |
| Brown-headed Cowbird | summer; resident (along Pacific coastline) | early May | mid-Aug | Trees | Grasslands | Granivore | Ground Forager |
| Bushtit | resident | early Feb | early Aug | Trees | Scrub | Insectivore | Foliage Gleaner |
| California Scrub-jay | resident | early Jul | late Aug | Trees | Scrub | Ominvore | Ground Forager |
| Canada Goose | resident | early Feb | Jul | Ground | Marshes | Granivore | Ground Forager |
| Chestnut-backed Chickadee | resident | early Apr | late Jun | Cavities | Forests | Insectivore | Foliage Gleaner |
| Dark-eyed Junco | resident | mid-May | late Aug | Ground | Forests | Granivore | Ground Forager |
| Downy Woodpecker | resident | early May | late Jun | Cavities | Forests | Insectivore | Bark Forager |
| Eurasian Collared-Dove | resident | year round | year round | Trees | Towns | Granivore | Ground Forager |
| European Starling | resident | late Mar | early Jun | Cavities | Towns | Insectivore | Ground Forager |
| Glaucous-winged Gull | resident (western WA); winter (western OR) | early Jun | late Aug | Ground | Shorelines | Ominvore | Ground Forager |
| Golden-crowned Kinglet | resident (western WA and OR); winter (eastern WA and OR) | late May | late Jul | Trees | Forests | Insectivore | Foliage Gleaner |
| Golden-crowned Sparrow | winter (western WA and OR); migrant (eastern WA and OR) | N/A | N/A | Ground | Scrub | Insectivore | Ground Forager |
| Great Blue Heron | resident | late Feb | mid-Jul | Trees | Marshes | Piscivore | Stalking |
| House Finch | resident | early May | late Jul | Trees | Towns | Granivore | Ground Forager |
| House Sparrow | resident | late Apr | early Oct | Cavities | Towns | Ominvore | Ground Forager |
| Lesser Goldfinch | accidental (WA); resident (western OR); summer (eastern OR) | early Apr | late Aug | Trees | Open Woodlands | Granivore | Foliage Gleaner |
| Mallard | resident | early Apr | early Sep | Ground | Lakes and Ponds | Ominvore | Dabbler |
| Mourning Dove | resident | early Jun | early Jul | Trees | Open Woodlands | Granivore | Ground Forager |
| Northern Flicker | resident | late Mar | early Jul | Cavities | Open Woodlands | Insectivore | Ground Forager |
| Orange-crowned Warbler | summer; resident (along Pacific coastline) | late Apr | late Jun | Ground | Forests | Insectivore | Foliage Gleaner |
| Osprey | summer; migrant (pockets in eastern WA and OR) | late March | early Sep | Trees | Oceans | Piscivore | Aerial Dive |
| Pine Siskin | resident (abundance subject to irruptions) | late Mar | mid-Aug | Trees | Open Woodlands | Granivore | Foliage Gleaner |
| Purple Finch | resident | early Jun | early Jul | Trees | Forests | Granivore | Foliage Gleaner |
| Red-breasted Nuthatch | resident | early Apr | late May | Cavities | Forests | Insectivore | Bark Forager |
| Red-winged Blackbird | resident | mid-Apr | early Jun | Shrubs | Marshes | Insectivore | Ground Forager |
| Rock Pigeon | resident | early Apr | early Jul | Buildings | Towns | Granivore | Ground Forager |
| Ruby-crowned Kinglet | winter (lowland); summer (montane) | early May | early Aug | Trees | Forests | Insectivore | Foliage Gleaner |
| Rufous Hummingbird | summer | early May | early Jul | Trees | Open Woodlands | Nectarivore | Hovering |
| Song Sparrow | resident | late Mar | late Aug | Shrubs | Open Woodlands | Insectivore | Ground Forager |
| Spotted Towhee | resident | early Apr | late Aug | Ground | Scrub | Ominvore | Ground Forager |
| Steller's Jay | resident | mid-Mar | early Jul | Trees | Forests | Ominvore | Ground Forager |
| Tree Swallow | summer | late Apr | late Jul | Cavities | Lakes and Ponds | Insectivore | Aerial Forager |
| Violet-green Swallow | summer | early May | early Aug | Cavities | Open Woodlands | Insectivore | Aerial Forager |
| White-crowned Sparrow | winter; resident (along Pacific coastline) | early Apr | late Jul | Ground | Scrub | Insectivore | Ground Forager |
| Wilson's Warbler | summer | early Jun | early Jul | Ground | Scrub | Insectivore | Foliage Gleaner |
| Yellow-rumped Warbler | resident (western WA and OR); summer (central WA and OR); mixed elsewhere | early May | mid-Jul | Trees | Forests | Insectivore | Foliage Gleaner |
